# Liver Transplantation for Alcoholic Liver Disease

**Published:** 2003

**Authors:** Abhinandana Anantharaju, David H. Van Thiel

**Affiliations:** Abhinandana Anantharaju, M.D., is a fellow, and David H. Van Thiel, M.D., is a professor of medicine and surgery and director of Liver Transplantation and Hepatology. Both are associated with the Liver Transplant Program in the Division of Gastroenterology, Hepatology, and Nutrition, at Loyola University Medical Center, Maywood, Illinois

**Keywords:** alcoholic liver disorder, organ transplantation, liver, patient assessment, patient compliance, comorbidity, cardiomyopathy, pancreatitis, malnutrition, hepatocellular carcinoma, AODR (alcohol and other drug related) structural brain damage, bone mineral density, hepatitis C virus, AOD abstinence, alcohol use test treatment outcome, quality of life, AODD (alcohol and other drug dependence) relapse, predictive factor, patient monitoring, medical ethics

## Abstract

In many patients, long-term heavy drinking leads to chronic liver disease, liver failure, and even death. Orthotopic liver transplantation (OLT) is the only definitive treatment for end-stage liver disease, including alcoholic liver disease (ALD). Because of a shortage of donor organs, OLT for ALD patients remains controversial out of concerns that patients may resume drinking, thereby harming the transplanted organ. Therefore, transplant centers conduct careful screening procedures that assess patients’ coexisting medical problems and psychosocial status to identify those patients who are medically most suited for the procedure and who are most likely to remain abstinent after OLT. Studies assessing the outcomes of ALD patients after OLT found that the survival rates of the transplanted organ and the patient were comparable to those of patients with nonalcoholic liver disease and that relapse rates among the ALD patients were low. Similarly, ALD patients and patients with other types of liver disease had comparable rates of compliance with complex medication regimens after OLT. Enhanced efforts to identify risk factors for relapse among OLT candidates with ALD and to target interventions specifically to those patients who are at high risk of relapse may further improve patient outcome and enhance the acceptance of OLT for alcoholic patients in the general population.

Population-based surveys indicate that 68 percent of adult Americans drink at least one alcoholic beverage per month. About 10 percent consume more than two drinks per day, which is a commonly used definition of “heavy drinking” (Hoofnagle et al. 1997). However, substantial differences exist in the prevalence of heavy drinking among population subgroups. For example, 18 percent of men but only 3 percent of women are classified as heavy drinkers, and heavy drinking is more common among Whites than among African Americans or Hispanics. Heavy drinking and its consequences are important public health problems, as illustrated by the following statistics:

- Five percent of the deaths occurring annually in the United States (approximately 100,000 per year) are either directly or indirectly attributable to alcohol abuse (Hoofnagle et al. 1997).
- Only about 10 percent of all drinkers account for 50 percent of the total alcohol consumption in the United States per year (Li 1997).
- About 13.8 million people in the United States meet the diagnostic criteria for alcohol abuse or dependence specified in the fourth edition of the American Psychiatric Association’s *Diagnostic and Statistical Manual of Mental Disorders* (Grant et al. 1994).
- About 15 percent of U.S. alcoholics eventually will develop alcoholic liver disease (ALD), a broad spectrum of liver injuries—ranging from asymptomatic fatty liver (i.e., steatosis) or abnormalities of liver enzymes to end-stage liver disease—that result from alcohol ingestion. Women in general show greater susceptibility to ALD than men, and African Americans show greater susceptibility than Whites.
- Among heavy drinkers, liver disease is highly prevalent. Thus, 90 to 100 percent of heavy drinkers have steatosis, 10 to 35 percent have alcohol-induced inflammation of the liver (i.e., alcoholic hepatitis), and 8 to 20 percent have alcoholic cirrhosis (McCullough 1999).
- The 5-year and 10-year survival rates for patients with alcoholic cirrhosis are 23 percent and 7 percent, respectively (McCullough 1999). These rates are significantly worse than survival rates for patients whose cirrhosis was not caused by alcohol.

Alcohol consumption is one of the leading causes of chronic liver disease in the United States and worldwide. In Western countries, alcohol is the major causative factor in about 50 percent of deaths from end-stage liver disease (McCullough 1999). To date, liver transplantation (also known as orthotopic^1^ liver transplantation [OLT]) is the only definitive treatment for end-stage liver disease. However, OLT for patients with ALD continues to be controversial because of the ever-increasing demand for donor organs and the inadequate rate of organ donation, combined with concerns that alcoholic patients might relapse to drinking, thereby damaging the transplanted liver.

This review discusses the patterns and controversies relating to liver transplantation in patients with ALD. After providing some historical perspective and summarizing the current status of OLT in these patients, the article discusses elements of the pretransplantation evaluation that can help identify suitable patients for the procedure. Outcomes for ALD patients who have received liver transplants are reviewed, and the ethical issues surrounding this procedure in alcoholic patients are discussed. This article concludes by summarizing future research directions that might improve the outcomes of liver transplants in alcoholics and thereby resolve some of the ethical concerns.

## Historical Perspective and Current Status of OLT in Alcoholic Patients

### Historical Perspective

Before the National Institutes of Health (NIH) Consensus Conference on Liver Transplantation in 1983, OLT rarely was performed in patients with ALD. The Consensus Conference concluded that ALD is an appropriate indication for OLT if the patient is judged likely to abstain from alcohol after transplantation (Lucey 2001). This conclusion, which was adopted by many transplant centers, led to an increase in the number of transplants performed for ALD. A report by Starzl and colleagues (1988) made the most convincing argument for OLT for ALD patients, demonstrating that 73 percent of ALD patients who had received a liver transplant still were alive 1 year after the procedure, and that only 3 percent of those patients had relapsed to alcoholism. Based on these findings, in 1991 the Health Care Financing Administration (HCFA) identified ALD as one of the seven conditions for which it approved payment for OLT, but the HCFA recommended a “significant” period of abstinence for alcoholics before undergoing the procedure as well as the availability of a reasonable social support system.

To identify alcoholic patients suitable for OLT, Beresford and colleagues (1990) proposed a selection method that included measures of the likelihood of abstinence, such as the extent to which alcohol dependence was recognized by the patient and his or her family, the patient’s degree of social stability, and the nature and extent of lifestyle changes conducive to long-term abstinence. Using this selection method, Lucey and colleagues (1992) reported on a multidisciplinary collaboration of transplant hepatologists, surgeons, and psychiatrists that identified psychosocial predictors of long-term sobriety and compliance after OLT in alcoholics. (These predictors are summarized in the University of Michigan Alcoholism Prognosis Scale, which is discussed later.) These researchers reported that ALD patients judged suitable using these criteria had outcomes after OLT that were similar to outcomes for transplant patients with non-alcohol-related liver disease (non-ALD). People who were judged suitable for OLT included patients with severe end-stage liver disease without an available alternative therapy, who showed a clear understanding of the risks and benefits of the procedure, had a favorable psychiatric assessment including acceptance of alcoholism, and had favorable prognostic factors for future sobriety.

The minimal listing criteria established by the United Network for Organ Sharing (UNOS) in 1996 do not include an absolute requirement for a 6-month period of abstinence before ALD patients are listed as candidates for OLT (UNOS 1996). Furthermore, a 1996 NIH workshop on OLT for patients with ALD concluded that liver transplantation provides a good outcome in alcoholic patients and that relapse rates after OLT were lower if the patients had successfully completed conventional alcohol rehabilitation programs prior to OLT (Hoofnagle et al. 1997).

ALD now is widely accepted by many transplant centers as a valid indication for OLT, provided the transplant team caring for the patient can reasonably expect him or her to remain abstinent after the transplant.

### Current Status

According to the UNOS database, 41,734 liver transplants using organs from dead donors (i.e., cadaveric transplants) were performed in the United States between 1992 and 2001 (UNOS 2002). Of those, 12.5 percent were performed in patients with ALD, and 5.8 percent were performed in patients with ALD and a concurrent infection with the hepatitis C virus (HCV).^2^ This makes ALD the second most frequent indication (after HCV infection alone) for which OLT was performed during this period. As of November 2002, 17,646 patients in the United States were on the waiting list for a cadaveric liver transplant; of these, about 14.1 percent had ALD, and 6.2 percent had combined ALD and HCV infection.

Overall, the number of liver transplants performed annually for ALD has been relatively constant for many years (see the accompanying figure), but the number performed because of chronic HCV infection has increased annually, as has the number of liver transplants for combined ALD and HCV infection.

## Pretransplant Evaluation of Patients With ALD

To ensure the success of liver transplantation, ALD patients are required to undergo a thorough evaluation to determine whether they are suitable candidates. This evaluation addresses any coexisting medical problems that might influence the outcome of the transplant and includes a psychological evaluation to identify those patients who are most likely to remain abstinent and comply with the medical regimen after the procedure.

### Coexisting Medical Problems

Alcohol affects many organ systems in addition to the liver. For example, as described by Keeffe (1997) and Neuberger and colleagues (2002), alcohol abuse can lead to:

- Damage of the heart muscles (i.e., cardiomyopathy) and skeletal muscles (i.e., skeletal myopathy).
- Inflammation of the pancreas (i.e., pancreatitis).
- Malnutrition.
- Central and peripheral nervous system dysfunction.
- “Soft” bones that lack minerals for stability (i.e., osteopenia/osteoporosis).
- Cancers of the airways and digestive tract.

These conditions, particularly if they are severe, can complicate the management of patients with ALD both before and after OLT, and some may even be contraindications for OLT (Keeffe 1997). However, some of these alcohol-induced conditions (e.g., cardiomyopathy and acute pancreatitis) can be reversed by abstinence, and when such a reversal occurs, these conditions do not affect the decision on a patient’s suitability for a transplant.

The clinical approach to evaluating a patient for OLT also may be markedly altered by other disorders that can coexist with ALD, such as infection with hepatitis viruses, particularly HCV, and the presence of liver cancer. The impact of all these coexisting conditions is discussed in the sections to follow.

#### Cardiomyopathy

The exact prevalence of heart disease in patients with end-stage ALD is unknown (Keeffe 1997). Overall, cardiomyopathy is far more common in actively drinking alcoholics than in abstinent alcoholics (Campbell and Lucey 1996). In general, alcohol-related cardiomyopathy rarely is a reason for refusing liver transplantation (Lucey 2001). Anecdotal evidence suggests that coronary artery disease (CAD) may be more prevalent than cardiomyopathy in patients with ALD (Keeffe 1997). To identify either condition in liver transplant candidates, many transplant centers routinely assess cardiac function through noninvasive tests (e.g., electrocardiography, echocardiography, and stress tests) as part of their pretransplant evaluations (Neuberger et al. 2002). A more invasive technique, coronary angiography, uses X rays to visualize the structure of the heart and blood vessels following the injection of a contrast medium, and can identify more patients with CAD than the various noninvasive cardiac tests used (Keeffe 1997). Although CAD is not a reason to refuse a patient a transplant because it usually can be reversed by abstinence, the condition can create problems if it has not been identified prior to the OLT.

#### Skeletal Myopathy

Muscle damage occurs in up to 42 percent of alcoholic patients with ALD; 46 percent of actively alcoholic men show changes in muscle cell structure indicative of skeletal myopathy (Keeffe 1997). This condition is manifested as muscle weakness, muscle pain, and abnormal tests for muscle enzymes; the disorder results from a combination of alcohol’s direct effects on the muscles, malnutrition, and alcohol-related inflammation or degeneration of nerves (i.e., neuropathy) (Keeffe 1997). The presence of skeletal myopathy appears to depend on how much alcohol the person has consumed over his or her lifetime (Keeffe 1997; Campbell and Lucey 1996). In general, skeletal myopathy is not a contraindication for OLT, and severe myopathy is unusual in potential alcoholic OLT candidates.

#### Pancreatitis

Chronic inflammation of the pancreas is five times less common in people with ALD than in alcoholics without liver disease; the reasons for this difference are not known (Keeffe 1997). In general, pancreatitis is not considered a contraindication for liver transplantation; however, severe chronic pancreatitis can adversely affect the absorption of medications that prevent the immune system from rejecting the transplanted liver. Therefore, patients with pancreatitis may require closer monitoring for rejection of the transplanted organ as well as administration of higher doses of antirejection medications to achieve effective concentrations.

#### Malnutrition

Malnutrition occurs in many, if not all, patients with ALD. Causes of malnutrition include a poor diet; increased breakdown (i.e., catabolism) of carbohydrates, proteins, and lipids in the body; as well as impaired absorption of nutrients, interruption of the bile flow (i.e., cholestasis), reduced pancreatic function, bacterial overgrowth, and/or alcohol-induced injury to the intestinal mucosa (Matos et al. 2002). In particular, alcoholics commonly show deficiencies in various vitamins, including thiamine, which is essential for normal brain functioning. Therefore, alcoholics with ALD routinely should be prescribed thiamine and multivitamins. Severe malnutrition is associated with a poorer prognosis after OLT and may require postponement of the procedure until the patient has achieved a better state of nutrition (Matos et al. 2002). The nutritional status of OLT candidates can be improved by providing additional nutrition directly into the gastrointestinal tract (i.e., by enteral feeding). Moreover, nutritional support before and after the transplant can improve the clinical outcome after OLT (Matos et al. 2002).

#### Neurological Deficits

Chronic alcoholism may lead to neurological deficits through alcohol’s direct actions on the brain and nerve fibers, which can result in structural damage (Lucey 2001; Keeffe 1997). In patients with ALD, however, neurological deficits also can result from a condition called hepatic encephalopathy, which is caused by the damaged liver’s inability to remove substances from the blood that can interrupt brain function. In these patients, it is difficult to distinguish deficits resulting from alcohol’s direct effects on the brain from those resulting from hepatic encephalopathy. Severe neurological deficits may contraindicate liver transplantation because the patient may not be able to comply with post-transplant medication regimens and because OLT may not improve the patient’s quality of life significantly. Therefore, most transplant centers routinely perform brain-imaging analysis of OLT candidates to identify any structural damage that may exist before the transplant and which could affect the patient’s outcome after the transplant (Lucey 2001).

#### Abnormal Bone Structure

Patients with ALD are prone to bone loss because of impaired activity of the bone-producing cells; reduced activity of the ovaries or testes, which produce hormones regulating bone formation; reduced body mass index; and limited physical activity (Keeffe 1997). Between 10 and 42 percent of patients with ALD have a reduced bone density, which can lead to a condition called osteopenia (or, in severe cases, osteoporosis), which is characterized by bone softening, accompanied by weakness and susceptibility to fractures. Therefore, routine bone mineral density measurements and, in appropriate cases, blood tests assessing calcium metabolism and ovarian or testicular function are recommended in patients with ALD. Treatment with calcium and vitamin D (which regulates calcium metabolism) can improve bone mineral density in patients with ALD (Rouillard and Lane 2001). Other approaches used to improve bone mineral density in patients with non-ALD include administration of hormones to compensate for reduced ovarian or testicular activity as well as treatment with other compounds that influence calcium metabolism (i.e., calcitonin and biphosphonates) (Rouillard and Lane 2001). The effectiveness of these approaches in alcoholics, however, has not been studied specifically.

#### HCV Infection

About 20 to 30 percent of patients with ALD are infected with HCV (Keeffe 1997), and the rate of progression of liver disease and the long-term outcome are worse for these patients than for alcoholics not infected with HCV (Peters and Terrault 2002). In addition, the most commonly used treatment for HCV infection—an agent called interferon—is less effective in active alcoholics, probably because the antiviral activity of interferon is decreased in these patients (Peters and Terrault 2002). HCV infection in alcoholic patients also influences the outcome after liver transplantation; in fact, the transplanted liver is much more likely to be damaged by renewed HCV infection in these patients than by a relapse to alcohol abuse.

Patients with ALD who also are infected with the hepatitis B virus face challenges similar to those experienced by ALD patients with HCV infection.

In general, patients with liver disease resulting from alcohol abuse and coexisting viral infection appear to have a worse prognosis than patients with liver disease resulting from only one of these factors (Bellamy et al. 2001).

#### Liver Cancer

Patients with alcoholic cirrhosis have an increased prevalence of liver cancer (i.e., hepatocellular carcinoma, or HCC) (Peters and Terrault 2002; Stickel et al. 2002). These tumors can substantially influence the patient’s outcome after OLT because of the risk that they will recur. The presence of HCC itself is not a contraindication for OLT, because patients can have a reasonably good prognosis after OLT if they have only small tumors (≤ 5 centimeters [cm] in diameter) and/or fewer than four tumors of 3 cm or less each that have not spread to major blood vessels or outside the liver. Studies have found that cancers other than HCC (e.g., cancers of the airways and digestive tract or lymph node tumors) occur significantly more commonly in patients undergoing OLT for ALD than for non-ALD and are a major cause of illness and death late after OLT for ALD (Bellamy et al. 2001). To rule out the presence of a coexisting liver cancer, routine hepatic imaging studies are recommended as part of a pretransplant workup for all OLT candidates. Similarly, it is imperative that patients being considered for OLT undergo a thorough pretransplant screening for tumors outside the liver as well as a regular evaluation after the transplantation.

### Psychiatric Evaluation

For OLT to be successful in alcoholic patients it is essential that the patients remain abstinent after the transplant and comply with the demanding medical regimen (e.g., consistently take the necessary antirejection medications). Routinely conducting psychiatric evaluations before patients are included on the list of candidates for transplantation may identify those who are most likely to fail these criteria.^3^ In a survey using a five-point questionnaire, staff at 93 percent of transplant centers felt that a psychiatric evaluation was an important component in the pretransplant workup, and staff at 83 percent of the centers reported routinely using a psychiatrist or addiction specialist during the pre-transplant evaluation (Everhart and Beresford 1997). In most cases, the psychiatric evaluation includes assessments of the patient’s socioeconomic condition as well as of underlying psychiatric disorders, job status, number and duration of prior attempts at abstinence, and use of other drugs. During this evaluation, the psychiatrist routinely interviews both the patient and one or more family members, and estimates the risk of post-transplant alcohol relapse.

One measure that has been proposed to predict post-transplant sobriety is the University of Michigan Alcoholism Prognosis Scale (MAPS), which assesses a variety of factors, including:

- The patient’s and family’s recognition and acceptance of alcoholism.
- Four prognostic factors indicating sobriety, including involvement in activities that can substitute for drinking (e.g., sports), negative behavioral consequences of alcoholism, presence of hope/self-esteem, and availability of social relationships.
- Social stability factors, such as a stable job, residence, and marriage, or living with another person.

Data on the effectiveness of the MAPS are equivocal, however. In a prospective study, patients identified as suitable OLT candidates based on their MAPS scores had a low incidence of pathological drinking 3 years after liver transplantation (Beresford et al. 1992). Conversely, a retrospective study conducted 5 years after ALD patients had received transplants showed that their pretransplant scores did not predict continued sobriety (Lucey et al. 1997).

Some researchers consider an abstinence period of 6 months prior to OLT a predictor of long-term abstinence (Beresford and Everson 2000; Weinrieb et al. 2000). Some transplant programs and insurance companies insist on an absolute 6-month period of abstinence before a patient with ALD can be listed for liver transplantation. This 6-month rule remains controversial, however, and appears to be arbitrary. Some studies favoring the 6-month rule have demonstrated that patients who are abstinent for less than 6 months have a greater relapse rate (Beresford and Everson 2000; Weinrieb et al. 2000), but these studies only examined short periods of time, included only a small number of patients, and did not include control subjects. In contrast, many retrospective and prospective studies have demonstrated that the 6-month rule does not predict long-term sobriety after OLT (see table 1). As a result, the current minimal listing criteria for liver transplantation proposed by UNOS do not require a 6-month period of abstinence before listing ALD patients for liver transplantation.

As an alternative to the 6-month abstinence requirement for predicting abstinence after OLT, Yates and colleagues (1998) proposed a High Risk Alcoholism Relapse (HRAR) scale, which is based on the patient’s history of heavy drinking, usual number of drinks, and number of prior alcoholism inpatient treatment episodes. Initial studies have demonstrated that patients with low HRAR scores had a low relapse rate and could be deemed eligible for transplant without a pre-OLT 6-month period of abstinence (Yates et al. 1998). A subsequent study by the same research group, however, showed that the HRAR scale had little ability to predict continued sobriety after OLT (DiMartini et al. 2000).

## Outcomes for ALD Patients After OLT

When assessing the long-term outcome for patients receiving OLT or any other kind of transplant, researchers and clinicians evaluate numerous factors in addition to the survival of the patient, including how long the transplanted organ continues to function (i.e., graft survival) and the patient’s quality of life. Out of concerns that ALD patients may resume drinking after OLT and thereby damage the transplanted liver, investigators frequently assess graft survival in these patients. These assessments have found that the graft-survival rate in patients with ALD is comparable to that of patients with non-ALD (see table 2) (UNOS 2002). This finding suggests that the ALD patients are not more likely to relapse (or that their alcohol consumption may not be likely to damage the transplanted liver). In fact, the 1- and 3-year graft-survival rates in patients with ALD are above the average graft-survival rates for all diagnoses for which OLTs are performed (see table 2). Moreover, the presence of a concurrent HCV infection does not appear to alter the 1-, 3-, and 5-year graft-survival rates in patients with ALD. However, a study by Neuberger and colleagues (2002) demonstrated that patients undergoing OLT for combined ALD and HCV infection were more likely to develop hepatic fibrosis than were patients with either ALD or HCV infection alone. Additional survival rates reported by various groups of investigators are summarized in table 1.

A few retrospective studies have been performed in abstinent patients who underwent OLT for ALD. These patients’ livers subsequently were removed and examined for the presence of hepatitis caused by alcohol. Again, the presence of hepatitis appeared to have no impact on outcome, with the survival and relapse rates of these patients comparable to those of patients with alcoholic cirrhosis alone (Lucey 2002). No specific studies have assessed OLT survival rates and sobriety in patients with acute alcoholic hepatitis.

### Quality of Life

The term “quality of life” encompasses various factors that influence a patient’s subjective well-being, such as medical status, social status, employment status, or relationships. Overall, the physical and psychological outcomes for ALD patients after OLT appear similar to those of non-ALD patients (Pereira et al. 2000). However, patients who relapse to alcohol use after receiving transplants have poorer post-transplant scores on quality-of-life measures than patients who do not relapse (Coffman et al. 1997).

ALD patients in general have problems keeping a job and fulfilling their job requirements (i.e., they have low levels of occupational functioning). OLT can ameliorate these problems to a certain extent. Nevertheless, an analysis combining the findings of several studies (i.e., a meta-analysis) demonstrated that the employment status of ALD patients both before and after transplantation is dismal (Bravata et al. 2001). Before transplantation, 29 percent of ALD patients and 59 percent of non-ALD patients were employed. At 3 years after the OLT, employment rates for non-ALD patients had increased substantially (i.e., to 80 percent), whereas employment rates for ALD patients had increased only marginally (i.e., to 33 percent). Furthermore, no associations were found between alcohol use and employment status after OLT or between pre- and post-transplant employment and sobriety. With all these findings it is important to note, however, that the employment status reported in many studies is based on self-reports, which have substantial limitations (i.e., respondents may not always answer truthfully or may not accurately recall the information).

### Relapse to Alcohol Use

As mentioned previously, an important concern in selecting alcoholic candidates for OLT and evaluating the outcome of the procedure is the likelihood of a relapse to alcohol use after the transplant. The definition of an alcohol relapse is controversial, varying from any use of alcohol after OLT to alcohol abuse resulting in physical and social consequences or rehospitalization for alcoholism (Fuller 1997). Although any alcohol use after OLT should be viewed as serious because it is the earliest indicator of high risk for the long-term viability of the graft (Beresford 1994), not all relapses may be harmful to the transplanted liver and the patient. The occasional use of small amounts of alcohol (i.e., a “slip”) is not considered harmful and should not be treated punitively (Fuller 1997). These slips may not progress to an overt relapse that is potentially harmful to the new liver.

Because of the differing definitions of relapse, the reported relapse rates vary widely across studies (see table 1), whereas the rates of graft dysfunction resulting from alcohol relapses are more consistent regardless of the definition of relapse used. Furthermore, the transplanted liver is rejected at a similar rate in both abstinent and nonabstinent alcoholic patients (Campbell and Lucey 1996). This rejection reaction can occur if the patient does not consistently take necessary antirejection medications. Studies have found that among patients receiving OLT for ALD, the overall rate of non-compliance with the antirejection medications is as high as 16 percent (Berlakovich et al. 2000). However, alcohol relapses per se do not appear to influence the patients’ compliance with their medication regimen.

Interestingly, a meta-analysis found that ALD and non-ALD patients reported similar rates of alcohol use at 6 months (4 percent and 5 percent, respectively) and 12 months (17 percent and 16 percent, respectively) after OLT (Bravata et al. 2001), although heavy drinking was more common in patients who had undergone liver transplantation for ALD. At 7 years after OLT, 32 percent of ALD patients reported drinking some alcohol (Bravata et al. 2001). As previously mentioned, continued alcohol use after OLT puts the patient at risk for renewed ALD. Studies have found, however, that from a purely biological perspective, recurrent ALD is less prevalent and less severe after OLT than recurrent liver disease from other causes (e.g., reinfection with a hepatitis virus) (Bellamy et al. 2001).

The reported relapse rate is influenced not only by the different definitions of relapse but also by the method used to identify a relapse. The most useful identification method appears to be a clinical interview conducted by a transplant psychiatrist or a questionnaire interview by an assistant (DiMartini et al. 2001). Biochemical markers (e.g., alcohol levels in the blood, urine, or breath, or tests for the presence of certain enzymes) as well as regular liver biopsies are less effective at identifying relapses (Neuberger et al. 2002; DiMartini et al. 2001; Campbell and Punch 1997). Relapse rates are highest during the initial 6 months after the transplant and decline after this period (Platz et al. 2000). About 95 percent of all relapses occur in the first 2 years after OLT.

### Predictors of Relapse After OLT

Most patients with ALD are less severely dependent on alcohol than patients attending alcohol clinics (Howard and Fahy 1997), possibly because patients who do not exhibit symptoms of severe alcohol dependence are at greater risk of developing ALD because they can sustain continuous alcohol consumption over many years (Wodak et al. 1983). The patient’s premorbid social stability and Alcoholics Anonymous attendance record before OLT are important determinants of sustained abstinence after the procedure (Howard and Fahy 1997; Beresford 1997). Such factors can be assessed prior to a transplant using measures such as the Strauss-Bacon and Skinner indices, but few transplant centers report using these indices (Beresford 1997).

Another factor influencing relapse risk is the presence of other psychiatric disorders. The prevalence of preexisting psychiatric disorders in ALD patients is unknown. The few studies conducted in this patient population appear to show a higher rate of major psychiatric disorders among ALD patients than in the general population (Beresford 1997). However, major depressive disorders or schizophrenic conditions, which would indicate a greater risk of relapse after OLT, occur only rarely (Howard and Fahy 1997; Beresford 1997). The presence of post-traumatic stress disorder also increases the risk of alcohol relapse.

Coexisting dependence on drugs other than alcohol also is associated with higher rates of alcohol relapse. Studies have found that although 30 to 50 percent of patients who are dependent only on alcohol achieve sustained abstinence after alcoholism treatment, only 10 percent of patients who used more than one drug achieved abstinence (Beresford 1997).^4^ A prolonged period of documented abstinence from all drugs can indicate a low risk of relapse. Conversely, multiple failed attempts at alcohol abstinence before OLT are considered an indication that the prognosis for sustained sobriety after the transplant is poor (Beresford 1997).

Based on long-term studies of alcoholism remissions and relapses, Vaillant (1995) proposed four prognostic factors that indicate a favorable outcome: the patient’s involvement in activities that can substitute for alcohol, a caring relationship with another person, a source of hope or improved self-esteem, and negative behavioral reinforcement for subsequent drinking (e.g., the development of acute pancreatitis). When at least two of these factors are present, the patient is likely to remain abstinent for 3 or more years. If none of these factors or only one of them applies, the patient is likely to relapse within 2 years.

Individual transplant centers also assess long-term abstinence among their patients. Based on their findings, UNOS developed the following guidelines for considering individual alcoholic patients for liver transplantation (UNOS 1996; Vaillant 1995):

- A few months of sobriety as a test of short-term compliance (UNOS does not require a 6-month period of abstinence).
- Presence of a supportive social network at home.
- Absence of comorbid risk factors.
- A clinical impression that the patient has been compliant in the past.

Some transplant centers have used contingency contracting as a method to improve long-term abstinence (DiMartini et al. 2002). With this approach, the patient and the center enter a formal agreement that specifies the consequences of certain actions of both parties. No studies to date have assessed the efficacy of this strategy, however.

Several investigators have proposed additional risk factors for an alcohol relapse (also see the textbox). A study by Platz and colleagues (2000) suggested that these risk factors include being female, having a poor social environment, having poor personal stability as assessed by a psychologist, and completing less than 6 months of abstinence. DiMartini and colleagues (2001) identified a history of other drug use, a family history of alcoholism, and previous experience with alcoholism treatment as risk factors associated with a higher incidence of alcohol relapse. These investigators did not find any relationship between a prior psychiatric disorder and abstinence at 6 months after the transplant.

**Predictors of Relapse in Patients With Alcoholic Liver Disease Following Liver Transplantation**

Psychiatric comorbidity
Social instability
Prior rehabilitation attempts
Lack of involvement with other activities
Poor self-image
Associated chronic illness
Abuse of more than one drug
Lack of coordinated care

### Types of Liver Damage During an Alcohol Relapse

Although it is not necessary to take a tissue sample (i.e., biopsy) of the liver to make a diagnosis of recurrent ALD in patients who have relapsed to alcohol use, researchers have examined changes in liver structure (i.e., histological changes) that occur in these patients. These analyses found that the histological features of recurrent ALD that affect the transplanted liver are similar to those of ALD in the native liver (Lee 1997). The major histological changes seen with recurring ALD in transplant patients are steatosis, which is found in 83 percent; steatosis accompanied by inflammation (i.e., steatohepatitis), found in 10 percent of cases; fibrosis in certain areas of the liver, which occurs in 28 percent of patients; and cirrhosis, found in 23 percent of patients. Other less commonly seen changes include enlarged mitochondria^5^ in 14 percent of cases, excess levels of iron (i.e., siderosis) in the liver cells in 24 percent of cases, and interruption of the bile flow within the liver. These changes are not specific for ALD, and the physician therefore must exclude the presence of other diseases (e.g., viral infection).

## Management of Alcoholic Patients After an OLT

With the exception of patients who are dependent on other drugs in addition to alcohol, ALD patients do not have a higher incidence than non-ALD patients of pre- or postoperative psychiatric problems that would necessitate additional treatment (Howard and Fahy 1997). However, ALD patients at high risk for relapse should be followed closely, and regular psychiatric followup should be considered in such cases.

To date, no controlled studies have evaluated specific treatment methods for managing relapse after liver transplantation. The alcoholism treatment approaches used in the general population are probably applicable to these patients as well, with close monitoring by the transplant psychiatrist/psychologist and physician. Several studies have demonstrated that the involvement of a transplant psychiatrist or psychologist both before and after OLT reduces the alcohol relapse rate after transplantation (Lucey et al. 1992; Berlakovich et al. 1994, 2000).

Several investigators have attempted to study the effectiveness of motivational enhancement therapy in patients who relapse after liver transplantation (DiMartini et al. 2002). The results of such studies are unclear, however, because few patients enroll in these studies and because other problems are associated with the care of such patients.

Another approach to achieving abstinence in alcoholic patients, administering the medication disulfiram, which serves to deter people from drinking by causing unpleasant effects when combined with alcohol, is not recommended in patients after OLT because disulfiram has toxic effects on the liver.

## Ethical Issues Associated With OLT for ALD Patients

Although most people in the population consume alcohol at least occasionally, alcoholism and the diseases caused by it continue to carry a stigma among the general public. This is true particularly for ALD, and no other alcohol-induced organ damage is viewed so negatively. Many people have a bias against liver transplants in alcoholics, resulting at least in part from the continued organ shortage and ever-increasing demand for donor organs, which necessitates rationing of the donor organs. For example, some people consider ALD a self-inflicted disease and therefore propose that ALD patients have lower priority on transplant waiting lists. This attitude is reflected in an opinion poll conducted in Great Britain, which showed that both the general public and family physicians believed that alcoholic patients should have a lower priority than others for OLT (Neuberger et al. 1998). Similar results were reported in Oregon, where the general public was asked to allocate priorities for 714 disorders or treatments. The respondents rated the priority of OLT for non-ALD patients at a moderate level (364 out of 714), but gave a very low priority (695/714) to OLT for ALD (McMaster 2000). Transplant psychiatrists and psychologists, however, have a more favorable opinion on OLT for alcoholics. For example, in a survey of these health care professionals from 14 academic liver transplant centers in the United States, a consensus favored offering further alcoholism treatment to patients who continued to use alcohol rather than refusing OLT outright (Snyder et al. 1996).

Researchers and clinicians originally thought that ALD patients would have poorer graft and patient survival rates than non-ALD patients, but such a difference has never been documented. Neither has the assumption of high relapse rates in ALD patients been confirmed. Relapse rates following OLT are lower than in patients undergoing alcoholism treatment, and serious relapses that adversely affect the transplanted liver or the patient are uncommon (see table 1). In contrast, patients who receive OLT because of an infection with hepatitis B or C viruses always experience disease recurrence and have an increased likelihood of losing the transplanted liver primarily because of this recurrence. Another concern, that patients with ALD would not be able to comply with the anti-rejection medication regimen, also has not been confirmed. Graft-rejection rates are similar for patients transplanted for ALD and those transplanted for other types of liver disease, which indicates comparable rates of compliance with the antirejection medications. Finally, it was anticipated that ALD patients would utilize more resources, thereby incurring higher costs, than non-ALD patients, but again this assumption never has been corroborated by research evidence (Campbell and Lucey 1996).

In contrast to these negative assumptions regarding the outcome of OLT in ALD patients, many clinicians argue that ALD is an excellent indication for liver transplantation. For example, the overall improvement in the status of patients with ALD after OLT, including the improvement in productivity, supports considering such patients for liver transplantation. Moreover, the long-term costs of OLT and the subsequent management of the alcoholic patient probably are lower than the costs of managing alcoholism and ALD without transplantation. This assumption is based on the observation that only 22 percent of all alcohol-dependent people seek help within any 1-year period, and fewer than one drug abuser in eight receives formal treatment for alcohol or other drug dependence (Keeffe 1997).

The suitability of ALD patients for OLT also must be considered in the context of the three principles of organ and tissue allocation identified by UNOS (Ethics Committee, UNOS 1992). First, *the principle of medical utility* requires that organs be allocated to those patients who are likely to experience the maximum medical benefit. This requirement is well satisfied by patients with ALD. Second, *the principle of justice* requires that equal respect and concern be given to all patients in need of an organ or tissue. This principle generates some ethical questions regarding the potential wastage of organs by transplantation in patients who may “voluntarily” revert to alcoholism. Third, *the principle of autonomy* requires that the personal choices of the patients be respected. In this regard, the UNOS Ethics Committee holds the view that a person’s past behavior (including alcohol consumption) should not be considered as an exclusion criterion for OLT.

There is no one-to-one correlation between alcoholism and ALD (Benjamin 1997). Only a minority of alcoholics develop ALD and may require OLT; conversely, many patients diagnosed with ALD do not meet the criteria for alcoholism. However, the indiscriminate allocation of a donor liver to an alcoholic who may relapse and thus endanger the function of the transplanted organ is not justifiable to the general public. It is important to remember that the general public makes up the donor pool and may be discouraged from organ donation by a policy of equal transplantation for alcoholics and nonalcoholics. Thus, clinicians must make an effort to identify OLT candidates among the ALD patients who are at low risk for relapse. Moreover, additional education of the public is necessary to displace the stigma for OLT in alcoholics and to increase organ donation.

## Future Directions

Because outcome after liver transplantation, particularly the risk of relapse, is such an important concern in patients with ALD, identifying the factors that could indicate the most suitable candidates for OLT is a highly desirable goal of research (Li 1997). The following lines of inquiry show promise in this regard:

- Studies have identified genetic characteristics that influence alcohol intake. Additional in-depth analyses are needed to determine the specific influences of these genes on the development of ALD or on the risk of alcohol relapse after OLT.
- Genetic factors may determine a person’s susceptibility to developing liver damage after alcohol consumption or to becoming alcohol dependent. Such possible factors should be investigated further, because transplanting a new liver might alter or negate the genetic influence. For example, a patient with a high susceptibility to ALD might not be as susceptible to ALD after OLT even if he or she returned to heavy drinking because the new liver would be governed by its own set of genetic factors. Conversely, if genetic factors determining alcohol metabolism by the liver play a role in maintaining alcohol dependence, then OLT might “cure” the addiction.
- Researchers must develop better means of identifying ALD patients who are at risk of relapse after OLT. With better identification methods, transplant centers could focus their resources on this rather small group of patients before OLT in an effort to prevent subsequent relapses. Furthermore, research assessing various treatment programs may identify those approaches that best improve abstinence rates after OLT.
- Currently no definite blood tests (i.e., biochemical markers) can identify relapses after OLT. Isolated elevations in a liver enzyme called gamma-glutamyl transpeptidase without concomitant elevation of another enzyme, called alkaline phosphatase, may serve as a surrogate marker of relapse (Pappo et al. 1995). Further research directed at identifying a marker that can indicate abstinence over a period of time would be valuable for monitoring the patients’ drinking behavior before and after OLT.
- Investigators must further evaluate the outcome of liver transplantation in patients with severe alcoholic hepatitis; currently these patients rarely are considered for an OLT (because they usually do not have enough time to prove sobriety).

## Conclusions

OLT currently is the only definitive treatment for liver failure, including ALD. Because of the shortage of donated organs, however, OLT in patients with ALD remains controversial, mainly out of concerns that the transplanted liver could be “wasted” on a patient who eventually relapses to drinking, thereby damaging the transplanted liver. To address this concern, transplant centers generally perform a multidisciplinary screening procedure before the transplant to identify psychosocial predictors of relapse and select suitable ALD patients for OLT. Higher survival rates and lower relapse rates than expected in ALD patients after OLT have encouraged many transplant centers to reevaluate their criteria for these patients. As a result, many transplant centers currently do not require that ALD patients complete a 6-month abstinence period before being placed on a transplant list. Nevertheless, future studies should focus on identifying patients at risk for relapse, so that preventive and therapeutic interventions can be selectively targeted to these patients. The ethical debate regarding the justification of OLT in patients with ALD continues, although this subject is less controversial than in the past. Further education of the public regarding the outcomes of liver transplantation in ALD patients should help eliminate the stigma and misapprehensions associated with ALD in the context of OLT and could increase organ donation rates.

## Figures and Tables

**Figure 1:**
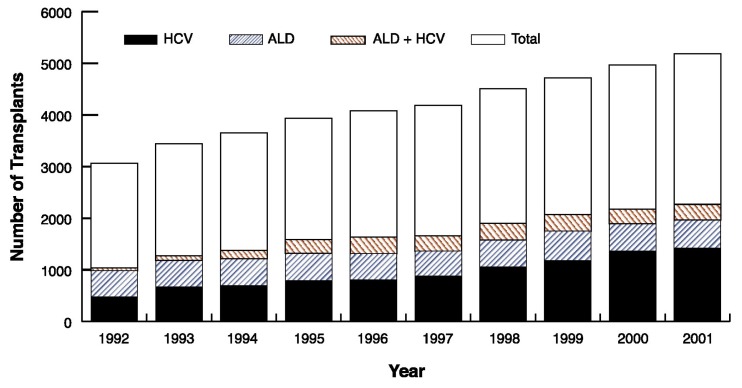
Liver transplantation for alcoholic liver disease (ALD) and hepatitis C (HCV), 1992–2001. SOURCE: United Network for Organ Sharing (UNOS) registry, 1988–2001. Public data from UNOS/OPTN scientific registry (http://www.unos.org). Accessed December 2002.

**Table 1:** Studies Reporting Outcomes of Orthotopic Liver Transplantation (OLT) in Patients With Alcoholic Liver Disease

Study*	Period	No. of Patients	No. of Patients Lost to Followup	Months of Followup After OLT	Patients Employed (Part-Time/Full-Time) (%)		Used 6-Month Abstinence Criteria?	Survival (%)				Patients Who Relapsed (%)	Research Method Used	Patients Who Experienced Graft Dysfunction Because of Relapse (%)	Deaths Related to Relapse (%)
	
					Before OLT	After OLT		1-yr	2-yr	3-yr	4-yr				
Starzl et al.	1980–87	41	11	12–36	N/A	N/A	No	73	-	-	-	3	Retrospective	3	3
Kumar et al.	1982–88	73	21	25	0	54	No	74	-	-	-	11.5	Phone interview	2	2
Bird et al.	1980–89	24	6	4–84	N/A	94	No	66	-	-	-	22	Retrospective	17	0
Stevens et al.	1985–90	10	-	1–24	N/A	N/A	No	-	-	-	-	0	Retrospective	0	0
Knechtle et al.	1984–90	41	30	N/A	63	33	No	83	-	-	71	13	Psychiatric interview	0	0
Goldstein et al.	1985–91	41	4	6–66	N/A	N/A	Yes	86	-	-	72	13.5	Retrospective	N/A	N/A
Gish et al.	1988–91	29	0	12–41	N/A	80	No	93	-	-	-	21	Prospective, combined	-	0
Osorio et al.	1988–91	43	0	7–38	62	59	Yes	100	-	-	-	19	Mailed questionnaire	N/A	0
Berlakovich et al. (1994)	1982–93	58	14	33	48	89	No	71	66	-	63	31	Clinic interview	16	3.4
Howard et al.	1987–92	20	20	12–72	N/A	N/A	N/A	79	-	-	-	95	Extensive interviews	10	N/A
Raakow et al.	1988–94	78	0	0.5–64	N/A	99	No	96	96	-	85	22	Retrospective	-	2.6
Gerhardt et al.	1985–91	67	26	36–96	N/A	N/A	No	90	84	82	76	49	Phone interview	N/A	4.5
Tringali et al.	1988–90	103	45	18–46	33	40	No	-	-	-	-	21	Retrospective, combined	N/A	N/A
Zibari et al.	1986–92	42	0	N/A	0	76	No	74	71	71	-	7	Retrospective, combined	0	0
Lucey et al. (1997)	1987–91	59	9	6–89	N/A	N/A	No	80	-	-	77	34	Combined	N/A	2
Foster et al.	1986–94	84	21	28–70	N/A	N/A	No	79	75	-	-	21	Combined	17	5
Anand et al.	1987–94	39	0	7–63	N/A	N/A	No	79	-	-	79	13	Clinic interview	2.6	0
Everson et al.	1988–96	68	6	< 90	N/A	N/A	N/A	91	-	-	-	30	Phone interview	9.7	6.5
Stefanini et al.	1986–96	18	7	< 118	0	73	Yes	75	75	-	75	27	Retrospective, combined	27	N/A
Fabrega et al.	N/A	44	0	20–59	N/A	N/A	Yes	-	-	-	-	18	Urine alcohol measurement	7	N/A
Tang et al.	1987–96	71	15	NA	8	52	No	83	80	-	-	48	Clinic interview	N/A	1.4
Pageaux et al.	1989–94	53	0	42	N/A	30	No	75	69	67	62	32	Clinic interview	4	2

* For full citations of these studies, see References.

**Table 2:** Percentage of Liver Transplant Patients in Whom the Transplanted Organ Was Still Functional at 1, 3, and 5 Years After the Procedure, Listed According to the Underlying Causes of the Patient’s Liver Disease

Underlying Cause of the Liver Disease	Number of Patients	Survival (%)		

		1-Year	3-Year	5-Year
Hepatitis C virus (HCV) infection	9,525	77.3	67.5	61.0
Alcoholic liver disease (ALD)	6,527	77.1	68.9	60.8
Acute hepatic necrosis*	3,546	66.9	59.4	54.2
Other postnecrotic* causes	3,500	72.7	63.8	56.6
Primary sclerosing cholangitis*	3,469	83.0	77.8	74.1
Primary biliary cirrhosis*	3,345	80.3	75.5	71.3
ALD + HCV infection	2,402	79.8	67.9	61.7
Metabolic disease	1,958	77.3	71.6	67.3
Autoimmune* liver disease	1,381	78.8	71.5	66.0
Liver cancer (hepatocellular carcinoma)	1,187	68.1	51.2	37.5
All Causes	46,940	74.5	67.4	62.7

* Necrosis is tissue death occurring in groups of cells; cholangitis is an inflammation of the bile ducts; biliary cirrhosis is an inflammation of the liver resulting when bile flow through small ducts in the liver is obstructed; autoimmune diseases are those conditions in which the body’s immune system erroneously attacks the body’s own cells.

SOURCE: United Network for Organ Sharing (UNOS) registry, 1988–2001. Public data from UNOS/OPTN scientific registry (http://www.unos.org). Accessed December 2002.

## Footnotes

1 Orthotopic means “in the normal or usual place.”

2 Infection with HCV (as well as with other related viruses, such as the hepatitis B virus) can lead to chronic inflammation of the liver (i.e., hepatitis), which eventually can cause complete liver failure and death.

3 Currently, ALD patients get on transplant lists later than patients with nonalcoholic liver disease because of the requirement for an evaluation of future abstinence.

4 The failure of investigators to distinguish between alcohol dependence and multiple-drug dependence in patients with ALD is one of the reasons for the variable relapse rates after OLT.

5 The mitochondria are membrane-enclosed components of the cell that are responsible for most of the cell’s energy production.
